# Identifying barriers and facilitators to reporting and disclosing sexual violence (SV) for undergraduate students in Canadian and American universities: a scoping review protocol

**DOI:** 10.1186/s13643-025-02942-9

**Published:** 2025-10-24

**Authors:** Karen Kennedy, Emily MacLeod, Jason Lee, Crystal Treige, Rebecca Jones, Rachita Narang, Virginia Gunn

**Affiliations:** 1 Cape Breton University, 1250 Grand Lake Road, Sydney, NS B1M1A2 Canada; 2 Unit of Occupational Medicine, Institute of Environmental Medicine, Karolinska Institute, Nobels väg 13, 171 77 Stockholm, Sweden

## Abstract

**Background:**

Canadian and American undergraduate universities grapple with gender-based violence, notably sexual violence (SV), primarily affecting women in their initial four months of university. However, an estimated 90% of survivors/victims do not report assaults, exacerbating psychological trauma and impacting resource allocation and the implementation of effective prevention strategies. Survivors/victims’ reluctance to report also denies them access to university accommodations and resources, impacting health and well-being. Immediate risks of SV include physical and emotional trauma, while long-term consequences encompass mental health conditions, educational setbacks, and economic losses.

**Objective:**

This study aims to comprehensively review and synthesize literature on underreported SV in Canadian/American universities. Through assessment, we seek to understand the strengths and gaps in current knowledge. Collaborating with experts, we aim to co-create recommendations for revising colonialized policies and reporting procedures, potentially eliminating barriers and enhancing facilitators. Our primary question focuses on identifying SV reporting barriers and facilitators for Canadian/American undergraduate survivors/victims. Given that a significant number of students at Canadian and American universities identify as Indigenous, African Canadian/American, International, and 2SLBGTQIAA + and we would like to know if the barriers and facilitators to reporting SV differ for these students, we added several sub-questions exploring intersections of race, culture, and gender. Addressing these knowledge gaps will inform policymaking, guide policy revisions, and improve reporting pathways, ultimately enhancing student safety and fostering violence-free Canadian and American campuses.

**Design:**

Databases inclusive of PubMED, CINHAL, PsycInfo, ERIC, and Web of Science as well as sources of grey literature were used to identify papers published between 2000 and 2024, from which we selected articles relevant to barriers and facilitators of reporting sexual violence in Canadian and American undergraduate universities.

**Results:**

We will employ the PRISMA flow chart for the systematic search, selection, and inclusion of evidence. Each source’s key characteristics will be summarized in the text, while detailed information will be organized in tables and appendixes. Specific data, aligned with our primary and sub-questions, will be charted, and a narrative synthesis will be formulated using the results table.

**Conclusions:**

The study’s results will offer a comprehensive overview of obstacles and factors influencing the reporting of sexual violence, highlighting variations based on cultural and sexual identities. Addressing these knowledge gaps will provide data for shaping recommendations, refining policies, and enhancing accessible reporting avenues. Ultimately, this contributes to improving student safety and fostering violence-free campuses in Canada and the United States (US).

## Introduction

### Rationale

Gender-based violence (GBV), specifically sexual violence (SV), continues to be a pervasive issue at Canadian and American universities that primarily impacts women in their first four months of attendance [3, 17]). A survey conducted in 2019 across post-secondary institutions in both countries revealed that 71% of students had witnessed or experienced an unwanted sexual incident [3]. Moreover, Boucek [2] estimates that a substantial 90% of survivors/victims choose not to report their assault, exacerbating survivor/victim psychological trauma [10]. In the university context, reporting and disclosure are separate and distinct concepts. Reporting SV at university typically involves formally notifying campus authorities about an incident. On the other hand, disclosing SV often refers to privately sharing one’s experience with a trusted individual or counselor without necessarily initiating a formal investigation. Access to resources and support can be facilitated when a survivors/victims report or disclose their assault, thereby providing an opportunity to lessen the negative effects of SV. Immediate risks associated with SV include physical injury, sexually transmitted infections (STIs), unwanted pregnancy, and emotional trauma [7], while long-term consequences encompass an elevated risk of experiencing mental health conditions, particularly post-traumatic stress disorder (PTSD), along with the loss of hope, agency, and an increased likelihood of suicidality [4, 6].

Noteworthy evidence points to several factors contributing to non-reporting among students, such as distrust, shame, secrecy, guilt, and embarrassment, concerns related to anonymity and confidentiality, fear of disbelief and retaliation, reluctance to prosecute, and financial dependence on the assailant [1, 2, 13, 15, 16]. Missed reporting opportunities prevent students from accessing university accommodations and resources, negatively impacting health and well-being [12]. Beyond the health risks listed above, SV detrimentally impacts educational achievement and degree progression [9], leading to subsequent economic losses related to time off, decreased performance, job loss, and an inability to work [11].

The Courage to Act Report (2019) developed by and for post-secondary institutions indicates the need to enhance SV reporting. While there is research discussing several barriers and facilitators of GBV, no systematic reviews have been conducted to capture the depth and complexity of factors related to underreporting [8]. As a result of this knowledge gap, our understanding of reporting is hindered. In addition, more evidence synthesis is needed to accurately unpack the complex interplay between SV reporting, gender, and culture of victims/survivors. Without this, it is difficult to gain a comprehensive understanding of the ways in which barriers and facilitators differ among subgroups given added vulnerabilities possibly created by various forms of oppression [5]. The results of this scoping review will provide data to inform recommendations for policymakers and guide the strengthening of policies related to reporting processes.

### Objectives

The primary aim of this scoping review is to identify barriers and facilitators to SV reporting and disclosure in US and Canadian universities, as identified in primary research studies. Our objective is to critically discuss the current state of knowledge surrounding this issue by identifying strengths and gaps in the literature. By integrating this synthesized knowledge with expert insights from our collaborators, we intend to co-create recommendations for revising existing policies and reporting procedures to reduce barriers and enhance facilitators for undergraduate students.

Our overarching review question focuses on uncovering barriers and facilitators to reporting SV for undergraduate survivors/victims at Canadian and American universities. We have also formulated sub-questions aiming to explore the intersections of race, culture, and gender to facilitate a deeper understanding of how barriers and facilitators to reporting SV may differ among student survivors/victims representing Indigenous, African Canadian/American, International, and 2SLBGTQIAA+ groups studying in Canadian and American universities. These sub-questions will provide a more nuanced and intersectional exploration of the unique challenges and support mechanisms present within diverse university communities.

Despite a general understanding of existing barriers, our review aims to address two specific research gaps: (1) a comprehensive synthesis of knowledge regarding the barriers and facilitators to SV reporting and (2) an exploration of how barriers and facilitators vary according to cultural and sexual identities. By addressing these gaps, our review will contribute valuable data and insights to inform policymakers, guide policy revision, and strengthen accessible pathways to reporting. Ultimately, these efforts aim to enhance student safety and foster violence-free Canadian and American campuses.

### Research questions

#### Main question

What are the barriers and facilitators to reporting and disclosing SV in Canadian and American undergraduate universities?

#### Sub-questions

Considering the diversity of students enrolled in universities in Canada and the USA, we have also formulated several review sub-questions aiming to explore the intersections of race, culture, and gender, hoping to facilitate a deeper understanding of the ways in which barriers and facilitators to reporting SV may differ among student survivors/victims representing Indigenous, African Canadian/American, International, and 2SLBGTQIAA+ groups.

- What are the barriers and facilitators to reporting and disclosing SV for Indigenous students in Canadian and American undergraduate universities?
- What are the barriers and facilitators to reporting and disclosing SV for African students in Canadian and American undergraduate universities?
- What are the barriers and facilitators to reporting and disclosing SV for international students in Canadian and American undergraduate universities?
- What are the barriers and facilitators to reporting and disclosing SV for 2SLBGTQIAA+ students in Canadian and American undergraduate universities?

## Methods

### Protocol and registration

This scoping review will be conducted in accordance with McGowan et al. [14] and Tricco et al. [18]. We will complete a rigorous systematic search of four health science electronic publication databases and grey literature. All findings will be reported using the Preferred Reporting Items for Systematic Reviews and Meta-Analyses extensions for Scoping Reviews (PRISMA-ScR) guidelines [14, 18]. The final protocol was registered prospectively with the Open Science Framework on 12 January 2023 (https://osf.io/wuckb/).

### Eligibility criteria

Due to human resource capacity related to linguistic ability, articles will be limited to English only. Eligible literature will include the following: (1) undergraduate students belonging to any gender who self-report experiencing SV while attending a post-secondary institution; (2) was conducted within Canada or the USA; and (3) published between 2000 and 2024. The scoping review will include primary research studies that utilize qualitative, quantitative, or mixed-methods study designs as well as a broad range of publications, including peer-reviewed articles, theses, and dissertations that identify or discuss SV reporting barriers and facilitators in higher education institutions (HEI). Relevant grey literature on the subject matter will also be included.

Literature will be excluded if (1) student survivor/victims studying above or below the undergraduate level; (2) have suffered abuse other than SV; (3) it is secondary research; (4) experienced an incident of SV not covered by institutional SV policies (e.g., SV that did not occur at a HEI or HEI sanctioned event); and (5) focuses on informal disclosures (e.g., disclosure to family or friends).

### Information sources

A scoping review, following the PRISMA-ScR guidelines [14, 18], will be performed to comprehensively understand the current literature and gaps. We registered the protocol with Open Science Framework. Searches will cover four academic databases (i.e., CINAHL, PsycINFO, ERIC, and PubMed), along with grey literature. We will use the Population, Intervention, and Context framework to guide our search and organize search terms. Our inclusion and exclusion criteria will guide study selection.

### Search

The search will be conducted by an experienced health sciences librarian who is a member of the research team. The strategy will be organized using the Population, Intervention, Context (PICo) framework. Relevant terms for undergraduate students, reporting of SV, and geography will be identified and used. The proposed search strategy for PubMed is outlined below. The PubMed search strategy will then be tailored to five additional databases: Cumulative Index to Nursing and Allied Health Literature (CINAHL), Education Resource Information Center (ERIC), PsycINFO, and Web of Science. Following the initial screening, a forward and backward review of the references from accepted article will take place. Grey literature will be obtained from Canadian and American governments, non-government organizations (NGO)s, community organizations, and university websites, as well as news articles using keywords university students, post-secondary students, sexual violence, sexual assault, report, and self-disclosure. All search results will be imported into Zotero® to remove duplicates and then placed into the Covidence® systematic review software for screening and data charting.

**Table:** 

Pub Med: Performed Feb 2, 2024	
Population	(university students or college students or undergraduate students)
Intervention	((disclo* OR self-disclos* OR self disclos* OR report* OR barrier* OR facilitator* OR abuse report OR self-report OR self report) AND (sex* harassment OR sexual coercion OR sexual assault OR sex* violence OR rape OR sex* crimes OR acquaintance rape OR sex* offence* OR unwanted sexual contact OR date rape OR dating violence, drug facilitated sex* OR drug-facilitated sex))
Context	(canada OR united states OR north america)
Filters	English, MEDLINE, 2000–2024
Results	765

### Selection of sources of evidence

Following a pilot screening of 30 studies, the titles and abstracts of potentially relevant literature will be screened independently by all members of the research team working in pairs using the inclusion and exclusion criteria. Piloting will be repeated as necessary until consistency in screening is achieved. The screening guidance document will be amended to reflect discussion incurred during the pilot phase. The remaining title/abstract screening will be completed following a successful pilot. The team will then progress to full-text review using the same process. Consensus will be reached through discussions with the principal investigator.

### Data charting process

Two reviewers will develop a structured data-charting form that the research team and study collaborators will vet. The tool will also be piloted with a subset of references. The data items abstracted from the full-text articles will include participant demographics (e.g., age, gender, race, culture, sexuality), type of SV (e.g., sexual assault, sexual harassment, sexual coercion), study characteristics (e.g., qualitative, quantitative, methodology, sample size), barriers to SV reporting, facilitators to SV reporting, university context (e.g., size, type), and article characteristics (e.g., author, year of publication, country of origin, funder, article type). The two reviewers independently charted the data, discussed the results, and continuously updated the data charting form in an iterative process. If a new category is identified during the charting process, the data charting form will be updated, and the studies already abstracted will be re-reviewed. We will provide the details of any modifications or updates in the final scoping review report.

This tailored data extraction tool, reflective of the scoping review questions, will be developed and piloted. The results will be presented in a structured narrative as well as tabular format, ensuring that the summary of results aligns with the review’s objectives and questions. The synthesis report will contain information about the gaps identified and a list of recommendations for revising policies related to SV reporting. We will integrate this synthesized knowledge with expert insight from collaborators to co-create recommendations for revisions to existing colonialized policies and reporting procedures, thus, potentially eliminating barriers and enhancing facilitators.

### Data items

We abstracted data on demographics (e.g., age, gender, race, culture, sexuality), type of SV (e.g., sexual assault, sexual harassment, sexual coercion), study characteristics (e.g., qualitative, quantitative, methodology, sample size), barriers to SV reporting, facilitators to SV reporting, university context (e.g., size, type), and article characteristics (e.g., country of origin, funder, article type). The data extraction tool includes distinct columns for the identification of barriers and facilitators for students studying in universities in Canada and the USA who identify as Indigenous, African Canadian/American, International, and 2SLBGTQIAA + groups.

### Synthesis of results

We will synthesize and present the results in narrative and tabular form. We will focus on barriers and facilitators to present them in the overall university context and consider the sub-questions guiding the study. Our primary purpose is to examine and consolidate existing literature regarding the underreporting of SV in Canadian and American universities, critically evaluate the current state of knowledge, and discern the strengths and shortcomings within the literature. We will also focus on equity within this context by addressing the sub-questions that are designed to spotlight various intersections involving race, sexuality, and culture.

## Results

### Selection of sources of evidence

We will use the PRISMA flow chart to describe the process of searching, selecting, and inclusion of evidence [18].

### Characteristics of sources of evidence

Pertinent characteristics from each source of evidence, accompanied by their references, will be outlined. A comprehensive summary will be presented in the text, while specific details for individual sources of evidence will be organized in tables and appendixes, as deemed appropriate.

### Critical appraisal within sources of evidence

We will not include a critical appraisal of our findings.

### Results of individual sources of evidence

Specific data will be charted according to our primary question and sub-questions. Relevant data from each source will be provided in an appendix.

### Synthesis of results

The table displaying the results of individual sources of evidence will be used to formulate a narrative as they relate to the review questions and objectives.

## Discussion

### Summary of evidence

In this scoping review, we will synthesize the results generally based on the major characteristics, barriers, and facilitators to reporting SV for Canadian/American undergraduate students. We will also present the characteristics, barriers, and facilitators according to our sub-questions that seek to highlight barriers and facilitators according to sub-populations (i.e., race, culture, and sexuality).

### Limitations

Our scoping review has some limitations. To make our review more feasible, we will include English only publications and grey literature was limited to Canadian and American government, NGO, community organizations, and university websites. Furthermore, the authors will not take into consideration articles pertaining to regions outside of Canada and the USA. Given that the search covers only articles focused on universities in Canada and the USA, we acknowledge that the findings re: barriers and facilitators to reporting SV will be limited to insights from these two countries only, thus missing out on a range of barriers and facilitators specific to other country contexts. Studies published beyond February 2024 and books and book chapters will not be included.

## Conclusions

A high-level summary of the identified data will be presented. Results of the study will provide a thorough compilation of information pertaining to obstacles and factors that encourage reporting of SV; and they will report the variations in barriers and facilitators based on cultural and sexual identities. Filling these knowledge gaps will consolidate data to shape recommendations for policymakers, assist in policy refinement, and bolster accessible avenues for reporting. This, in turn, will improve student safety and contribute to the establishment of violence-free campuses across Canada and the USA.

## Data Availability

Data available upon request from the corresponding author.
